# Advances in Developing CAR T-Cell Therapy for HIV Cure

**DOI:** 10.3389/fimmu.2020.00361

**Published:** 2020-03-10

**Authors:** Jinxin Qi, Chengchao Ding, Xian Jiang, Yong Gao

**Affiliations:** ^1^Department of Dermatology, West China Hospital, Sichuan University, Chengdu, China; ^2^Department of Microbiology and Immunology, The University of Western Ontario, London, ON, Canada; ^3^The First Affiliated Hospital, Department of Life Science and Medicine, University of Science and Technology of China, Hefei, China

**Keywords:** human immunodeficiency virus (HIV), acquired immune deficiency syndrome (AIDS), immunotherapy, chimeric antigen receptor-modified T cell (CAR T), HIV latent reservoir, HIV cure

## Abstract

Acquired immune deficiency syndrome (AIDS), which is caused by HIV infection, is an epidemic disease that has killed millions of people in the last several decades. Although combination antiretroviral therapy (cART) has enabled tremendous progress in suppressing HIV replication, it fails to eliminate HIV latently infected cells, and infected individuals remain HIV positive for life. Lifelong antiretroviral therapy is required to maintain control of virus replication, which may result in significant problems, including long-term toxicity, high cost, and stigma. Therefore, novel therapeutic strategies are urgently needed to eliminate the viral reservoir in the host for HIV cure. In this review, we compare several potential strategies regarding HIV cure and focus on how we might utilize chimeric antigen receptor-modified T cells (CAR T) as a therapy to cure HIV infection.

## Introduction

According to UNAIDS, more than 70 million people have been infected with the human immunodeficiency virus (HIV) and about 35 million people have died of HIV infection since this epidemic was first identified in the early 1980s. Globally, 37.9 million people were living with HIV at the end of 2018. The most effective and powerful therapy for HIV infection currently is combination antiretroviral therapy (cART), which has remarkably reduced morbidity and mortality and achieves durable suppression of plasma viremia below the limit of detection (1). The therapy has greatly extended life expectancy, turning HIV into a chronic disease that can be controlled instead of a death sentence, and has helped HIV-infected individuals live an almost normal life.

However, cART fails to cure HIV infection because of the existence of the latent viral reservoir, which is mainly a group of latently infected resting memory CD4+ T cells containing replication-competent HIV. All the cell types bearing the CD4 and its co-receptor (CCR5 or CXCR4) can be infected and become HIV latent reservoirs, including monocytes, macrophages, and dendritic cells (2). The HIV reservoir can exist in various compartments, such as peripheral blood, lymph nodes, the central nervous system, gut-associated lymphoid tissue (GALT), the genital tract, and any other tissues that contain HIV-infected cells (3). This latency does not express virus under the suppression of cART, but it can cause virus rebound once the therapy is interrupted (4). Thus, the persistence of HIV latency is regarded as the major obstacle to viral eradication (5–7). Meanwhile, as the half-life of HIV under effective cART is 44 months long (8, 9), more than 70 years of treatment is needed to achieve viral eradication. Hence, cART alone cannot get rid of the HIV reservoir, no matter how effective the drugs might be in controlling viral replication (10). Certainly, long-term use of cART brings up problems such as accumulative toxicity (11), drug resistance (12, 13), patient compliance, high cost, and even social problems like stigma. Additionally, chronic low-grade inflammation due to HIV infection continues even under the control of cART, which can accelerate aging, causing frailty syndrome at a younger age and a higher rate of comorbidity (11). So far, only two cases of HIV remission have been achieved in the past decades. These are Berlin and London patients who suffered from both hematologic malignancies and HIV infection, received CCR5Δ32/Δ32 allogeneic hematopoietic stem-cell transplantation (allo-HSCT), and achieved functional cure of both diseases. However, their success cannot be applied widely to HIV patients because of the high level of risk associated with marrow transplantation and there being limited suitable donors with CCR5Δ32/Δ32(14). Therefore, new strategies for HIV therapy that can achieve sterilization or functional cure need to be investigated. Treatment with chimeric antigen receptor-modified T cells (CAR T), a type of adoptive immunotherapy, has shown promising prospects for the therapy of B-cell malignancies (15–17). In parallel, HIV-specific CAR T cells have been designed for the treatment of HIV/AIDS. The first generation of HIV-specific CD4 receptor-based CAR was developed more than 20 years ago but was aborted because the resultant CAR T cell was susceptible to HIV infection and had negligible efficacy (18, 19). With the discovery of numerous potent anti-HIV broadly neutralizing antibodies (bNAbs) in recent years, bNAb-based CAR T therapy has been viewed as a potential strategy to cure HIV infection. Here, we provide an overview of recent studies on possible strategies for achieving HIV cure and mainly focus on the development, barriers, and future direction of CAR T therapy for HIV cure.

## Establishment of the HIV Latent Reservoir

The mechanism of formation of HIV latency in memory CD4+ T cells is still unclear but is likely to involve viral tropism and activation of CD4+ T cells. When resting CD4+ T cells are exposed to chemokines in tissue sites, they might become permissive to being infected by HIV (20). However, these cells are usually not easily infected due to their low expression of the co-receptor CCR5 unless they are activated to upregulate this essential element for viral entry (21). On the other hand, HIV replication in infected cells usually leads to cell death (22, 23). However, after infection by HIV, the activated CD4+ T cell could revert to a resting state, which results in minimal viral gene expression (24) and enables the host cell to escape from the immune attack and survive the viral cytopathic effects.

It is important to note that, in HIV infection, very few CD4+ T cells can transit from the activated state to the memory state, as most infected and activated CD4+ T cells are killed either by cytopathic effects or host immune targeting (24). It was determined that latently infected cells have an extremely low frequency in HIV+ individuals and that the virus yield is about 0.03–3.00 IU (infectious units) per million resting CD4+ T cells (9). However, during acute infection, the HIV replication level is very high, which allows for latency to develop shortly after infection (e.g., within 3 days of infection) (25).

## Stability of the HIV Latent Reservoir

One important characteristic of the HIV latent reservoir is its remarkable stability due to the persistence of HIV in the long-lived resting memory CD4+ T cells, which can undergo antigen-driven clonal expansion (5, 26). Evidence for clonal expansion of infected resting memory CD4+ T cells is provided by the predominant clones discovered in plasma, which remain unchanged over months to years in HIV patients under effective cART (27, 28). Analyzing the integration sites of HIV proviral DNA provides more direct evidence because HIV randomly integrates into transcriptionally active regions of the host genome of each infected resting CD4+ T cell clone (29–31). Several studies have identified identical HIV integration sites in multiple CD4+ T cells in HIV patients receiving effective cART (32–35). In addition, full-length proviral sequencing (32, 36) and *ex vivo* culture systems also provided evidence for clonal expansion of infected cells. Furthermore, three recent studies have all shown that 50–60% of the latent reservoir is made up of expanded clones at any given time (36–38).

Importantly, infected cells carrying defective proviruses appear to expand more than infected cells with active provirus, suggesting that defective proviruses produce fewer viral proteins inducing cytopathic effects or immune response (32). However, some studies show that clonal expansion also occurs in cells carrying replication-competent proviruses (34, 36–38), even though it could possibly lead to HIV gene expression in the cells and consequent viral cytopathic effects.

## Possible Strategies for HIV Cure

As mentioned above, cART cannot cure HIV infection due to the existence of the HIV latent reservoir. A number of strategies, including gene therapy, “block and lock,” and “shock and kill,” have been developed and tested in order to eradicate the HIV reservoir. However, despite inducing detectable latency reversal, these strategies have not yet been able to eliminate the latent reservoir completely.

### Gene Therapy

**Figure 1:**
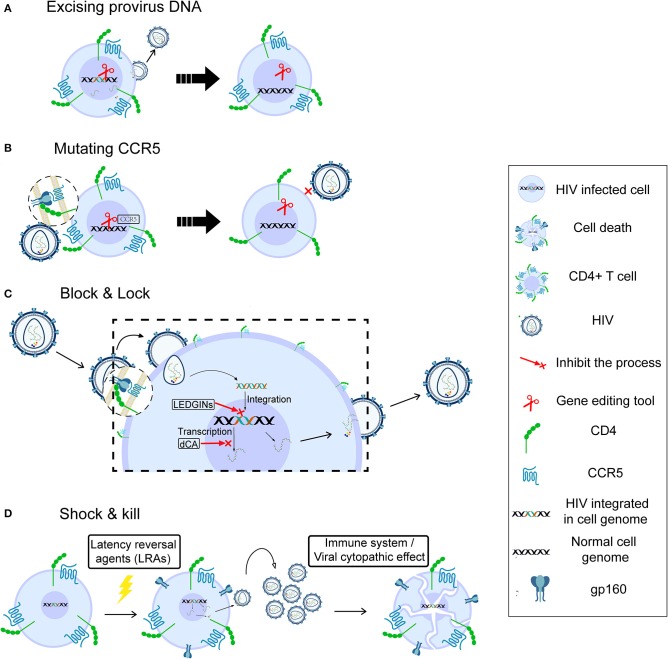
Possible strategies for HIV cure. Gene therapy for HIV cure by excising provirus DNA **(A)**, mutating CCR5 **(B)**, “block and lock” through silencing latent reservoir permanently **(C)**, and “shock and kill,” through activating HIV latently infected cells followed by immune destruction or viral cytopathic effects **(D)**.

There are mainly two strategies to cure HIV infection by using gene-editing tools, which are also commonly used for other diseases. The first is to remove the latent reservoir directly by excising the provirus (Figure 1A). Ebina et al. designed a CRISPR/Cas9 system targeting the HIV long terminal repeat (LTR) region to excise integrated HIV provirus from the latently infected resting CD4+ T cells. The result showed efficient editing in target sites and great loss of LTR-driven expression (39). Furthermore, the latest report indicated that HIV could be eliminated from cell and tissue reservoirs in sequential long-acting slow effective release ART (LASER ART) and CRISPR/Cas9-treated humanized mice (40). This first successful experiment using an animal model shows that gene therapy should be combined with precisely targeted treatment delivery to effectively block HIV viral growth and provirus integration. However, the safety of CRISPR-based gene editing in the context of the human gene therapy is largely unknown, and the ethical issues involving human genome manipulation must also be taken into account.

A second strategy for gene therapy is to stop new infection, aiming at functional cure. HIV enters a target cell with the help of CD4 and the CCR5 (41) or CXCR4 (42) co-receptor. A homozygous 32-bp deletion in the CCR5 gene can make individuals naturally resistant to CCR5-tropic HIV infection (43, 44) though still susceptible to virus targeting CXCR4 tropism (45). The success of the “Berlin patient,” the first case in which HIV sterilizing cure was achieved by transplantation of allogeneic donor CCR5Δ32 hematopoietic stem progenitor cells (HSPCs) (46), demonstrated that disruption of the CCR5 gene to prevent new infection could be a potential cure (47). However, it is unclear which part of the treatment of this case, the total body irradiation before each HSCT or the HSCT itself, contributed more to this long-term HIV remission (14). The second case, the “London patient,” also achieved HIV remission after a single allo-HSCT with homozygous CCR5Δ32 donor cells but did not receive any irradiation (14). This strongly supports the strategy of deleting the CCR5 receptor on the cell surface to cure HIV infection. Tebas et al. made CCR5 gene permanently dysfunctional in autologous CD4+ T cells through ZFN modification (Figure 1B), then reinfused the modified T cells into patients. During treatment interruption and resultant viremia, the decline in circulating CCR5-modified cells was significantly less than the decline in unmodified cells, and the blood level of HIV DNA decreased in most patients (48). Recently, Xu et al. reported successful transplantation and long-term engraftment of CRISPR/Cas9-edited, CCR5-ablated HSPCs in a patient with HIV infection and acute lymphoblastic leukemia (49). However, the percentage of CCR5 ablation in lymphocytes was only ~5%. Moreover, a recent study showed that the mortality rate of homozygosity for CCR5-Δ32 mutation is higher (~21%) than for the other genotypes before age 76 (50). Hence, it is necessary to pay more attention to the safety and risks of gene editing and the potential deleterious effect of CCR5 mutation at the individual level.

### Block and Lock

Although cART cannot suppress HIV replication completely, it reveals the possibility of curing HIV through silencing the latent reservoir permanently, known as the block & lock strategy (Figure 1C). The whole process, from entry to virus release, can be the target of this strategy. Lentiviruses, including HIV, prefer to integrate into the active transcriptional regions of host DNA (29, 51), indicating that integrase inhibitors may help block the HIV reservoir. Vranckx et al. used LDGEF/p75 inhibitors (LEDGINs), a kind of integrase inhibitor that can inhibit HIV integrase from interacting with LEDGF/p75 host cofactor, to retarget HIV integration, resulting in the provirus becoming more refractory to reactivation even by using latency-reversing agents (LRAs) (52). Mousseau et al. showed that the Trans-activator of transcription (Tat) protein inhibitor didehydro-cortistatin A (dCA), an analog of the natural product cortistatin A, could bind to the trans-activating response (TAR)-binding domain of Tat and selectively inhibited Tat transactivation of the HIV promoter. Importantly, dCA abrogated viral production from stable reservoirs, reduced residual viremia during cART (53), and greatly diminished the capacity for virus reactivation (54). According to these results, the inclusion of a Tat inhibitor in current cART regimens may also contribute to the functional HIV cure.

### Shock and Kill

Initial attempts at viral eradication involved global T-cell activators such as IL-2 and IL-2 + anti-CD3 antibodies (55, 56). However, despite inducing detectable latency reversal, these strategies ultimately failed to reduce the latent reservoir size and were associated with significant side effects due to massive cytokine release (57). In the last few years, HIV cure research has focused on the “shock and kill” strategy, which is aimed at attacking the HIV reservoir directly. This strategy could effectively expose viral reservoirs to a combination of cART and immune-mediated destruction or even eliminate the latently infected cells through viral cytopathic effects (vCPE). The goal of the “shock” is to reactivate viral replication in infected resting CD4+ T cells by LRAs in order to induce “kill” through attack by the immune system or active viral production (Figure 1D). There are many kinds of LRAs, for example, Protein kinase C (PKC) agonists (58), Histone Deacetylase inhibitors (HDACi) (59), and Histone Methylation inhibitors (HMTi) (60, 61). The most promising LRAs at this time are non-specific Histone Deacetylase inhibitors (HDACi), as they can acetylate the histone of integrated proviral promoters *in vitro*. Vorinostat, Disulfiram, and Romidepsin have been tested in clinical studies as candidate HDACi to induce viral replication, while the activated immune response would be expected to “kill” cells producing HIV (62–65).

The discovery that some of the latently infected CD4+ T cells are HIV-specific inspired another approach to reverse HIV latency (66, 67). By using an HIV vaccine providing a near-complete representation of viral quasispecies, such HIV-specific latently infected cells might be reactivated (68), and at the same time, cytotoxic T Lymphocyte (CTL) priming as well as the “kill” part of “shock and kill” strategies could be enhanced (57). A latency-reversing intervention could induce HIV expression indirectly by involving other cells. For instance, the TLR-7 agonist GS-9620 shows the ability to indirectly induce HIV expression in CD4+ T cells, probably through IFN-γ release from plasmacytoid dendritic cells (69). However, some reports indicate that these strategies have been unable to significantly impact the HIV reservoir size in patients (70, 71). Additionally, there is evidence to suggest that certain strategies may also adversely affect immune responses (72, 73). Thus, extensive research is being conducted to build more powerful “kill” in order to improve the strategy, including broadly neutralizing antibodies and immune checkpoint inhibitors (74).

## CAR T Cell Therapy

**Figure 2:**
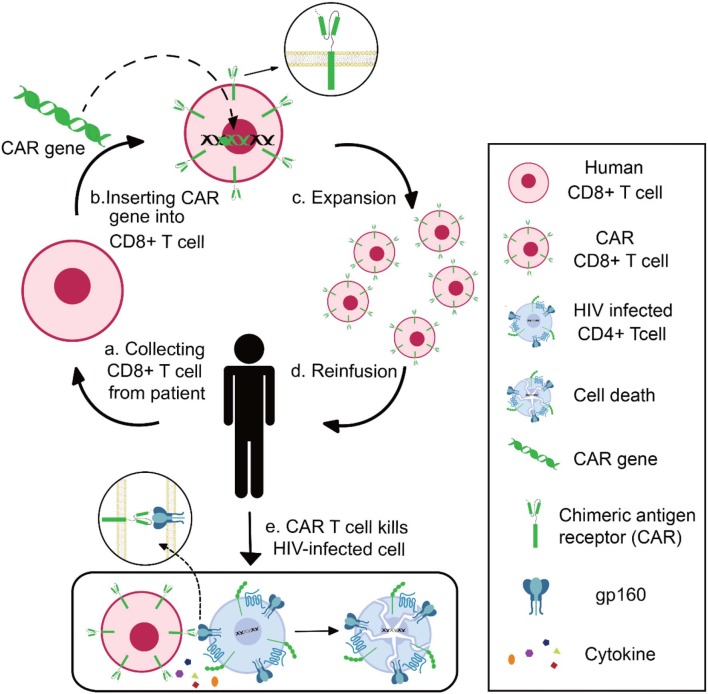
Schematics of CAR T-cell therapy for HIV infection. Collecting CD8+ T cell from HIV patient (a), inserting CAR genes into CD8+ T cells *in vitro* (b), expansion and functional identification of CAR T cells (c), reinfusing the HIV-specific CAR T cells into patients (d), and CAR T cells killing HIV-infected cells (e).

Chimeric antigen receptor (CAR) contains three domains: an extracellular domain to specifically bind antigens, a transmembrane portion to anchor the receptor, and an endo-domain to transfer signals (75). The extracellular domain is a single-chain fragment variant (scFv) derived from the variable domain of antibodies or receptors. The endo-domain being used is CD3ζ, a signal-transduction component of the T-cell antigen receptor (76). With these characteristics, researchers can design CAR to recognize specific antigens and activate CAR-expressed effector-cells (77). In practice, researchers could generate and expand CAR T cells from patients' blood, followed by reinfusion of CAR T cells into the patients (78). Take HIV-specific CAR T as example: CD8+ T cells are collected from HIV patients and transduced with CAR genes; after *in vitro* verification of the anti-HIV specificity and effectiveness, the functional HIV-specific CAR T cells are reinfused into patients to kill HIV-infected cells (Figure 2).

Four generations of CARs have been developed for the treatment of diseases since the chimeric antigen receptor was first presented in 1989. The first generation contains the CD3ζ-chain as the typical signal structure (75). The second generation adds additional costimulatory molecules like CD28, 4-1BB (CD137) to the signaling domain to improve the effector cells' proliferation, persistence, cytotoxicity, and sustained response (79–81). The third generation has two costimulatory molecules and outperforms the second generation in terms of cytotoxicity and long-term survival (82). The latest generation of CAR-T cells, also known as T cell redirected for universal cytokine-mediated killing (TRUCK), adds IL-12 gene into the CAR cassette; thus, CAR expression is accompanied by the release of IL-12. This structure enhances T-cell activation and attracts and activates innate immune cells to eliminate antigen-negative targeted cells in the targeted lesion (83).

### Success of CAR T Therapy in Cancer

Recently, by using autologous CD19-specific CAR-modified T cells, researchers have achieved complete remission in patients with hematologic malignancies like chronic lymphocytic leukemia (84), acute lymphoblastic leukemia (ALL) (85), diffuse large B-cell lymphoma, and follicular lymphoma (86) who were refractory to immunochemotherapy and transplantation or relapsed after the treatment. These results showed the safety, efficiency, feasibility, and durable effect of CAR T therapy. In the field of anti-ALL, CAR T therapy achieved complete remission in 27 out of 30 patients with relapsed or refractory ALL. At 6 months, the event-free survival rate was 67%, while the overall rate of survival is 78%. Durable remissions up to 24 months were also observed (85). While CAR T therapy worked well in hematologic cancers, it has been less effective in the treatment of solid tumors, likely due to lymphocyte trafficking problems (87). Fortunately, this might not be a limitation for HIV, as the main latency pool of HIV consists of CD4+ T cells (6, 11–13).

### CAR T Therapy for HIV Cure

The CTL response is a key component of host immunity against HIV infection (88, 89). In elite controllers, a rare group of people who are able to control HIV replication by their immune system for a prolonged period without anti-HIV treatment (90), it is believed that their spontaneous viral control is mediated largely by CD8+ T-cell response (91, 92). In addition to its significant role in suppressing HIV replication during acute infection (93, 94), boosting HIV-specific CTL responses before viral activation by LRAs could lead to rapid and effective killing of infected cells (57). On the other hand, due to this robust selective pressure, HIV quickly obtains mutations to escape CTL recognition (93, 95). It is reported that unless cART is started in the early stages of HIV infection, the vast majority (>98%) of latent viruses will carry CTL escape mutations (95). Studies also revealed that elite controllers have higher functional avidity and broader variant cross-reactivity of CTL responses when compared with non-controllers, indicating the critical importance of dealing with viral escape mutations for controlling HIV infection (96). Therefore, equipping CD8+ T cells with a CAR that is able to recognize various HIV antigens is a key for HIV cure. At present, the CD4 receptor (97) and bNAbs (98, 99) are used to construct anti-HIV CARs. CD4, which interacts with gp120 during HIV infection, has naturally high affinity to HIV. The CD4 receptor-based CAR T cell was demonstrated to have the same level of kinetics of lysis and efficiency of inhibition as naturally occurring CTL clones (100). Despite the fact that the CD4 receptor can fully neutralize all HIV isolates, the CD4 receptor-based CARs make the gene-modified T cells vulnerable to HIV infection.

Broadly neutralizing antibodies against HIV are found in ~20% of HIV-infected individuals. These bNAbs target HIV envelope glycoprotein (Env) and have the ability to neutralize most circulating HIV strains (101). Due to their affinity, potency, and breadth of anti-HIV neutralization, it is believed that developing bNAb-CAR for HIV cure would be effective. Hale et al. tested four types of bNAb-based CARs (PGT-128, PGT-145, VRC07-523, and 10E8). Co-culturing with a stably infected HIV-positive T cell line in the presence of ART, primary human T cells engineered with bNAb-based CARs showed specific activation and killing of HIV-infected cells (99). CTL can mediate infected cell lysis with the help of major histocompatibility complex class I (MHC-I) molecules, but HIV could downregulate the surface expression of MHC-I in infected cells to escape this immune response (102, 103). However, CAR T cells could overcome this viral escape mechanism, as the chimeric antigen receptor directly recognizes antigen without MHC I restriction (75, 104). Further, the long-term persistence of CAR T-cell therapy promises prolonged therapeutic benefit (85, 105). Scholler et al. reported that CD4 receptor-based CAR T cells have a decay half-life exceeding 16 years with stable levels of engraftment. As this group measured >500 patient-years of follow-up, their results also emphasized the safety of this therapy (105). One explanation for the long survival time of CAR T cells could be that a portion of these cells persist as functional memory T cells (88), whose life expectancy is much longer than that of effector T cells. Memory CAR T cells also promise to react rapidly and robustly, even if HIV infection reoccurs years later. Additionally, it was observed that CAR T cells persisted at high levels for at least 6 months in the cerebrospinal fluid (CSF) (106), a compartment that contains a significant but hard-to-reach reservoir of HIV (107). A recent study showed that primary T cells transduced with a multi-specific CAR (targeting both the gp120 CD4-binding site and the gp120 co-receptor–binding site) had the ability to potently reduce cellular HIV infection by up to 99% *in vitro* and >97% *in vivo* (108).

Two phase II clinical trials of CAR T therapy were carried out in 2000, using the same CAR design but treating two groups of patients with either undetectable viremia or active viral replication. In these trials, both CD4+ and CD8+ T cells were engineered with CD4ζ CAR including a CD28 costimulatory domain. The help provided by CD4-CAR CD4+ T cells was believed to contribute to the prolonged survival of engineered T cells in both studies (97, 109). In the trial with active viral replication, CAR T cells had high persistence in blood for the 8 week observation period (1–3% of peripheral blood mononuclear cells) and survived in 17 of 18 subjects for at least 1 year. In this trial, researchers also reported a >0.5 log mean decrease for at least 14 days in rectal tissue-associated HIV RNA, suggesting antiviral activity of these CAR T cells against this important tissue reservoir of HIV (109). In another trial in 2002, in which the plasma viral loads were <50 copies/ml, infusion of CD4-CAR T cells decreased HIV burden from baseline but caused no differences in the size of the viral reservoir (97). These trials confirmed the safety and feasibility of CD4-CAR T-cell therapy and suggested the necessity of enhancing *in vivo* expansion of chimeric receptor-modified T cells and optimizing *in vivo* function. Another two clinical trials of CAR T-cell therapy in HIV-positive patients under cART treatment are ongoing or in recruitment: one (NCT03240328) is testing a bNAb (VRC01)-based CAR, and the other (NCT03617198) is evaluating a CD4-CAR T cell modified by ZFN disruption of its CCR5 for HIV resistance.

### Obstacles and Solutions for Using CAR T Therapy in HIV

Even though it is predicted that anti-HIV CAR T therapy should be effective based on its performance *in vitro*, early clinical trials showed the safety of CAR T therapy but little efficacy *in vivo*. Besides, there are several obstacles or limitations as described below.

### Cell Expansion

**Figure 3:**
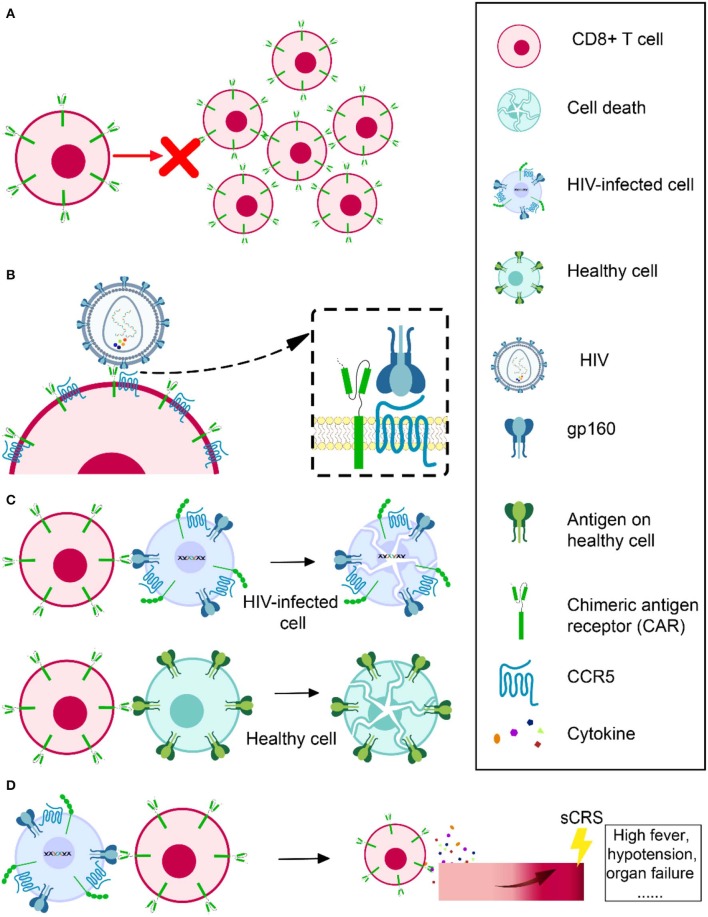
Obstacles in HIV-specific CAR T therapy development. Cell expansion and persistence *in vivo* **(A)**, susceptibility to HIV infection **(B)**, off-target effects **(C)**, and severe cytokine release syndrome **(D)**.

Several groups infusing expanded modified (110, 111) or even natural (112) CTL specific to HIV all showed that those cells died as a result of immune response or apoptosis in a very short time. Scholler et al. observed that CD4 receptor-based CAR T cells had no evidence of expansion and even persisted in high levels *in vivo* for decades (105). The problem of CAR T-cell expansion and persistence also occurred when applied in tumor treatment (113, 114). It is still unknown how much cell expansion and persistence contributes to the modest effect of CAR T cell *in vivo*. However, researchers consider it a possible direction for achieving a better outcome for CAR T therapy (Figure 3A). Sockolosky et al. utilized interleukin-2 (IL-2), a cytokine required for effector T cell expansion, survival, and function to help in CAR T-cell expansion. To avoid universal stimulation of effector T cells by IL-2, they engineered interleukin-2 (IL-2) cytokine-receptor orthogonal (ortho) pairs to interact with one another only, without interacting with their natural counterparts, while still transmitting native IL-2 signals. By introducing this engineered orthoIL-2Rβ into T cells, they selectively exploited the benefit of IL-2 with limited off-target effects and negligible toxicity (115). By using CRISPR/Cas9, Eyquem et al. directed CD19-specific CAR to the T-cell receptor α constant locus to help with CAR T-cell expansion. They reported that CAR expressed uniformly in human peripheral blood T cells, enhanced T-cell potency, performed tonic CAR signaling, and re-expressed following single or repeated exposure to antigen. Their results also showed a delay in effector T-cell differentiation and exhaustion (116). Fraietta et al. knocked down methylcytosine dioxygenase TET2 gene to improve the expansion and efficacy of CAR T cells and observed that, in one subject, 94% of CAR T cells likely originated from a single colony in which the TET2 gene was disrupted by the integrated CAR gene (117). Lack of antigen stimulation due to very low viral replication under cART might also contribute to the short-term persistence of infused CAR T cells. Therefore, in addition to a strong killing effect, the ideal shock and kill strategy using CAR T cells also needs to effectively reactivate latent reservoirs to produce virus, which will not only help CAR T cells to recognize the latently infected CD4+ T cells but will also help them to expand and achieve a better outcome.

### Susceptibility to HIV Infection

Another challenge is that CD4 CAR engineered CD8+ T cells are vulnerable to HIV infection (118), since CD8+ T cells have abundant CCR5 coreceptor on the cell surface (119) (Figure 3B). For this reason, Kamata et al. co-expressed two shRNAs, one targeting CCR5 expression and another targeting the HIV LTR, to prevent CD4 receptor-based CAR T cells from becoming HIV infected (120). Their results showed that over time, the number of shRNA-expressing CD4 receptor-based CAR T cells was higher than those without shRNA expression, suggesting that the susceptibility of CAR CD8+ T cell to HIV infection might partly account for its short persistence (120). Liu et al. constructed a novel bispecific CD4-CAR by adding a single-chain variable fragment of the 17b human monoclonal antibody, which can recognize a highly conserved epitope on gp120. Their CD4-17b CAR showed resistance to HIV infection, with higher potency of viral suppression than CD4 receptor-based CAR (18). To improve this bispecific CAR, the same group next substituted 17b with carbohydrate recognition domains (CRD) of a human C-type lectin that had been reported to interact with HIV gp120 mannose-containing glycans, including serum mannose-binding lectin (MBL), langerin, and liver/lymph node-specific intercellular adhesion molecule-3 grabbing non-integrin (L-SIGN). It was reported that the recognition by CRDs was a universal feature that would be hard for HIV to escape. They also emphasized that MBL, a soluble protein present in the circulation, should be safer, as immune reactivity against it should not target normal cellular self-protein. According to their results, this new bispecific CD4-lectin CARs enhanced potency and successfully prevented HIV infection (121). Using bNAb in CAR construction is another approach to prevent the susceptibility of engineered T cells. These bNAbs would not serve as a natural molecule on the cell surface to mediate HIV infection. Researchers using bNAb-based CAR have not reported any CAR T cells infected by HIV (98). Hale et al. even used a gene-editing tool to recombine a bNAb-CAR gene cassette into the CCR5 locus in order to destroy the CCR5 gene and ensure the engineered cells are HIV resistant and confirmed its feasibility and effectiveness (99).

### Off-Target Effects

Chimeric antigen receptors (CARs) could be constructed to specifically target HIV latently infected cells to achieve a cure. However, it is possible that CAR could also attack other healthy cells expressing the same or similar target antigen, which is known as an off-target effect (Figure 3C). For example, as CD19 is expressed on both normal and malignant B cells, B cell aplasia is inevitable when using CD19-based CAR T therapy to treat hematologic malignancies (84–86, 88). Fortunately, no evidence showed cytolysis of MHC II (CD4 counterpart)-expressing cell lines in CD4-CAR trials (18, 122). It is even safer to utilize bNAb-based CAR T therapy, because these bNAbs are specifically targeting HIV envelope glycoprotein, while CD4 may have more orthologs in nature. However, the ability of CAR to recognize its specific antigen without MHC I assistance raises another concern that it could recognize soluble antigen (e.g., cell-free or virion-associated HIV protein) and be activated to release cytokines toxic to surrounding tissues instead of specifically targeting HIV-infected cells.

### Severe Cytokine Release Syndrome

When CAR T cells are infused into a recipient, they will be activated through cognate interaction with their specific antigens. While they could specifically attack the target antigen-expressing cells, they might also result in a progressive systemic inflammatory process, known as severe cytokine release syndrome (sCRS), whose typical manifestations are usually high fever, hypotension, or even organ failure (Figure 3D). After the infusion of CAR T cells, CRS usually occurs within 1–14 days and has a duration of 2–3 weeks to reach full resolution, influenced by the product, clinical trial design, the individual being treated, and the intervention. IL-6 is a signature cytokine of CRS. CAR T cell-mediated clearance of cancer could trigger elevated IL-6 levels. The most important source of IL-6 during CRS is human monocytes. Therefore, getting rid of human monocytes or blocking IL-6 receptor with tocilizumab could prevent CRS. It was reported that tocilizumab rapidly reversed sCRS induced in a critically ill child by CAR T therapy (106). Corticosteroids are also helpful in some cases to control CRS. However, in contrast to tocilizumab, high doses of corticosteroids may impact the antitumor effect of the CAR T cells.

### Neurologic Toxicity

Neurologic events in CAR T therapy include encephalopathy, delirium, aphasia, focal deficits, and seizures. Neurologic toxicity is poorly understood but is considered to be associated with supraphysiologic levels of cytokines and CAR T cells crossing blood–brain barriers. Within the first few weeks of CAR T-cell therapy, neurologic events could occur following CRS or during resolution. Although both neurologic toxicity and CRS are related to high cytokine levels due to CAR T-cell activation, more severe systemic CRS can be a risk factor for neurologic toxicity. However, these two adverse events have independent definitions and need independent management. Tocilizumab was observed to have limited efficacy in resolving neurologic toxicity, possibly because of its poor central nervous system penetration (16, 123, 124). Thus, corticosteroids are used by some centers as first-line therapy for isolated neurologic toxicity (125–127). Optimized treatment algorithms and further research on mechanisms of CAR T-cell therapy-related neurologic toxicity are needed to achieve better management outcomes.

### Other New Development in CAR T Therapy

Beside the above-mentioned advantages and potential problems of CAR T therapy, there are some other important developments in HIV CAR T strategies, including improvements of CAR T in tumor therapy that might be useful for HIV cure. Leibman et al. optimized the construction of CD4 receptor-based CAR from the lessons of engineering CARs for hematologic malignancies (128). In terms of capacity to control HIV replication, their re-engineered CAR was at least 50-fold more effective than the original CD4 receptor-based CAR, a TCR-based approach, and bNAb-based CARs. By switching the MMLV-based gamma-retroviral vector to an HIV-based lentiviral vector and by switching the PGK promoter to an EF1α promoter, CAR surface expression was significantly augmented. In addition, the CD8α transmembrane (TM) domain took the place of the CD4 TM domain, which would be downregulated by HIV Vpu. The CD8α TM domain also promoted CAR dimerization, increased variability from the HIV cellular receptor, and enhanced the cytotoxicity of the resultant CAR. The substitution of the transmembrane domain also decreased the susceptibility of CAR CD8+ T cells to HIV infection. Next, different costimulatory molecules, including CD28, 4-1BB, CD28+4-1BB, OX40, ICOS, and CD27, were compared in culture. Experiments showed that ICOS, CD27, and 4-1BB co-stimulation impaired the suppression of HIV duplication. However, when compared with CD28-containing CARs, 4-1BB-containing CARs performed better in controlling early infection in the HIV prevention model and exhibited more durability in the treatment model. The authors concluded that the 4-1BB zeta signaling domain was optimal in HIV cure strategies for (1) acting rapidly to prevent HIV transmission, (2) durably preventing viral rebound, and (3) promoting T-cell survival in the absence of antigen.

It has been reported that, within B cell follicles where T follicular helper (Tfh) cells are located, the number of specific CTLs was too low to stop ongoing viral replication during chronic HIV infection. To solve this problem, Haran et al. co-expressed a potent bispecific anti-SIV CAR (rhCD4–MBL) with the B-cell follicle-homing chemokine receptor CXCR5 to direct CAR T cells. In *in vitro* migration assay, they showed that the CAR/CXCR5 T cells migrated to CXCL13, the ligand for CXCR5. Meanwhile, CXCR5 co-expression improved the concentration of CAR T cell in the B cell follicles in *ex vivo* tissues. They also showed that the co-expression of CXCR5 did not compromise the SIV-suppressive activity of the CAR T cell (129).

In addition to successful CD19 CAR T-cell therapy, there are also many other explorations of CARs for various tumor treatments, shedding light on the possible future applications of CAR T treatment for HIV infection. Wu et al. designed “ON-switch” CARs, whose function is controlled by a heterodimerizing small molecule, with the purpose of gaining more precise control over the timing, location, and dosage of T-cell activity, thus decreasing toxicity (130). CAR T strategies in current studies are all using patient-specific cells, the generation of which is prohibitively expensive. Researchers are now trying to develop universal CAR T cells that can adapt to multiple recipients. Torikai et al. took the very first step by using zinc-finger nucleases (ZFN) to eliminate the endogenous αβ-TCR in CAR T cells to avoid graft-vs.-host response. This study suggested that future studies should focus on inhibiting the attack of the recipient's immune system on allogeneic CAR+ T cells (131). Similarly, Hale et al. used homology-directed recombination (HDR) to target the CAR gene to the T cell receptor alpha constant (TRAC) locus, producing TCR-deficient CAR T cells. They then produced CCR5-negative anti-CD19 CAR T cells in the same way, which could be applied in treating HIV-associated B-cell malignancies. The most attractive feature of this HDR-generated CAR T cell was that the CAR gene integrated into a single, definitive target site, diminishing the risk of randomly insertional mutagenesis (132).

Besides CD8+ T cells, researchers have also investigated other immune cell types for alternative CAR therapy. For example, NK cells are considered to be promising candidates, because they do not require prior sensitization, they are not MHC-independent in nature, and they have shown less severe adverse effects since they are tightly controlled by inhibitory receptors. In addition, there are sufficient numbers of NK cells in peripheral blood and functional NK cell lines that can be used in clinical trials (133). Zhen et al. modified HSPCs with CD4zeta-based CAR and successfully differentiated them into functional T cells and NK cells upon transplantation into humanized mice. These modified HSPCs could continuously provide functional antigen-specific cells and suppress HIV replication, with possible resistance to virus infection (134).

## Conclusion

In summary, cART cannot eradicate HIV latency; therefore, new strategies for HIV cure are still needed and under development. There are three possible ways to achieve this goal: “block and lock,” “shock and kill,” and gene therapy. It is widely accepted that chimeric antigen receptor (especially bNAb-based CAR) engineered CD8+ T-cell therapy is a promising approach for curing HIV infection that is worth further exploration, even though there are limitations such as CAR T-cell expansion, persistence, off-target effect, and sCRS.

## Author Contributions

JQ, CD, and YG wrote sections of the manuscript. YG and XJ edited the manuscript.

### Conflict of Interest

The authors declare that the research was conducted in the absence of any commercial or financial relationships that could be construed as a potential conflict of interest.

## References

[1] Murphy EL Collier AC Kalish LA Assmann SF Para MF Flanigan TP. Highly active antiretroviral therapy decreases mortality and morbidity in patients with advanced HIV disease. Ann Intern Med. (2001) 135:17–26. 10.7326/0003-4819-135-1-200107030-00005 11434728

[2] Sadowski I Hashemi FB. Strategies to eradicate HIV from infected patients: elimination of latent provirus reservoirs. Cell Mol Life Sci. (2019) 76:3583–600. 10.1007/s00018-019-03156-8 31129856 PMC6697715

[3] Delannoy A Poirier M Bell B. Cat and mouse: HIV transcription in latency, immune evasion and cure/remission strategies. Viruses. (2019) 11:269. 10.3390/v11030269 30889861 PMC6466452

[4] Davey RT Jr Bhat N Yoder C Chun TW Metcalf JA. HIV-1 and T cell dynamics after interruption of highly active antiretroviral therapy (HAART) in patients with a history of sustained viral suppression. Proc Natl Acad Sci USA. (1999) 96:15109–14. 10.1073/pnas.96.26.15109 10611346 PMC24781

[5] Chun TW Stuyver L Mizell SB Ehler LA Mican JA Baseler M. Presence of an inducible HIV-1 latent reservoir during highly active antiretroviral therapy. Proc Natl Acad Sci USA. (1997) 94:13193–7. 10.1073/pnas.94.24.13193 9371822 PMC24285

[6] Finzi D Hermankova M Pierson T Carruth LM Buck C Chaisson RE. Identification of a reservoir for HIV-1 in patients on highly active antiretroviral therapy. Science. (1997) 278:1295–300. 10.1126/science.278.5341.1295 9360927

[7] Rong L Perelson AS. Modeling latently infected cell activation: viral and latent reservoir persistence, and viral blips in HIV-infected patients on potent therapy. PLoS Comput Biol. (2009) 5:e1000533. 10.1371/journal.pcbi.1000533 19834532 PMC2752194

[8] Finzi D Blankson J Siliciano JD Margolick JB Chadwick K Pierson T. Latent infection of CD4+ T cells provides a mechanism for lifelong persistence of HIV-1, even in patients on effective combination therapy. Nat Med. (1999) 5:512–7. 10.1038/8394 10229227

[9] Siliciano JD Kajdas J Finzi D Quinn TC Chadwick K Margolick JB. Long-term follow-up studies confirm the stability of the latent reservoir for HIV-1 in resting CD4+ T cells. Nat Med. (2003) 9:727–8. 10.1038/nm880 12754504

[10] Lorenzo-Redondo R Fryer HR Bedford T Kim EY Archer J Pond SLK. Persistent HIV-1 replication maintains the tissue reservoir during therapy. Nature. (2016) 530:51–6. 10.1038/nature16933 26814962 PMC4865637

[11] Guaraldi G Prakash M Moecklinghoff C Stellbrink HJ. Morbidity in older HIV-infected patients: impact of long-term antiretroviral use. AIDS Rev. (2014) 16:75–89. 24759453

[12] Boender TS Kityo CM Boerma RS Hamers RL Ondoa P Wellington M. Accumulation of HIV-1 drug resistance after continued virological failure on first-line ART in adults and children in sub-Saharan Africa. J Antimicrob Chemother. (2016) 71:2918–27. 10.1093/jac/dkw218 27342546

[13] Liu H Ma Y Su Y Smith MK Liu Y Jin Y. Emerging trends of HIV drug resistance in Chinese HIV-infected patients receiving first-line highly active antiretroviral therapy: a systematic review and meta-analysis. Clin Infect Dis. (2014) 59:1495–502. 10.1093/cid/ciu590 25053721 PMC4565655

[14] Gupta RK Abdul-Jawad S Mccoy LE Mok HP Peppa D Salgado M. HIV-1 remission following CCR5Δ32/Δ32 haematopoietic stem-cell transplantation. Nature. (2019) 568:244–8. 10.1038/s41586-019-1027-4 30836379 PMC7275870

[15] Brudno JN Kochenderfer JN. Chimeric antigen receptor T-cell therapies for lymphoma. Nat Rev Clin Oncol. (2018) 15:31–46. 10.1038/nrclinonc.2017.128 28857075 PMC12145160

[16] Fry TJ Shah NN Orentas RJ Stetler-Stevenson M Yuan CM Ramakrishna S. CD22-targeted CAR T cells induce remission in B-ALL that is naive or resistant to CD19-targeted CAR immunotherapy. Nat Med. (2018) 24:20–8. 10.1038/nm.4441 29155426 PMC5774642

[17] Ghorashian S Kramer AM Onuoha S Wright G Bartram J Richardson R. Enhanced CAR T cell expansion and prolonged persistence in pediatric patients with ALL treated with a low-affinity CD19 CAR. Nat Med. (2019) 25:1408–14. 10.1038/s41591-019-0549-5 31477906

[18] Liu L Patel B Ghanem MH Bundoc V Zheng Z Morgan RA. Novel CD4-based bispecific chimeric antigen receptor designed for enhanced anti-HIV potency and absence of HIV entry receptor activity. J Virol. (2015) 89:6685–94. 10.1128/JVI.00474-15 25878112 PMC4468509

[19] Zhen A Peterson CW Carrillo MA Reddy SS Youn CS Lam BB. Long-term persistence and function of hematopoietic stem cell-derived chimeric antigen receptor T cells in a nonhuman primate model of HIV/AIDS. PLoS Pathog. (2017) 13:e1006753. 10.1371/journal.ppat.1006753 29284044 PMC5746250

[20] Saleh S Solomon A Wightman F Xhilaga M Cameron PU Lewin SR. CCR7 ligands CCL19 and CCL21 increase permissiveness of resting memory CD4^+^ T cells to HIV-1 infection: a novel model of HIV-1 latency. Blood. (2007) 110:4161–4. 10.1182/blood-2007-06-097907 17881634

[21] Pierson T Hoffman TL Blankson J Finzi D Chadwick K Margolick JB. Characterization of chemokine receptor utilization of viruses in the latent reservoir for human immunodeficiency virus type 1. J Virol. (2000) 74:7824–33. 10.1128/JVI.74.17.7824-7833.2000 10933689 PMC112312

[22] Bleul CC Wu L Hoxie JA Springer TA Mackay CR. The HIV coreceptors CXCR4 and CCR5 are differentially expressed and regulated on human T lymphocytes. Proc Natl Acad Sci USA. (1997) 94:1925–30. 10.1073/pnas.94.5.1925 9050881 PMC20019

[23] Gornalusse GG Mummidi S Gaitan AA Jimenez F Ramsuran V Picton A. Epigenetic mechanisms, T-cell activation, and CCR5 genetics interact to regulate T-cell expression of CCR5, the major HIV-1 coreceptor. Proc Natl Acad Sci USA. (2015) 112:E4762–71. 10.1073/pnas.1423228112 26307764 PMC4553789

[24] Shan L Deng K Gao H Xing S Capoferri AA Durand CM. Transcriptional reprogramming during effector-to-memory transition renders CD4(+) T Cells permissive for latent HIV-1 infection. Immunity. (2017) 47:766–75. 10.1016/j.immuni.2017.09.014 29045905 PMC5948104

[25] Whitney JB Hill AL Sanisetty S Penaloza-MacMaster P Liu J Shetty M. Rapid seeding of the viral reservoir prior to SIV viraemia in rhesus monkeys. Nature. (2014) 512:74–7. 10.1038/nature13594 25042999 PMC4126858

[26] Brenchley JM Hill BJ Ambrozak DR Price DA Guenaga FJ Casazza JP. T-cell subsets that harbor human immunodeficiency virus (HIV) *in vivo*: implications for HIV pathogenesis. J Virol. (2004) 78:1160–8. 10.1128/JVI.78.3.1160-1168.2004 14722271 PMC321406

[27] Bailey JR Sedaghat AR Kieffer T Brennan T Lee PK Wind-Rotolo M. Residual human immunodeficiency virus type 1 viremia in some patients on antiretroviral therapy is dominated by a small number of invariant clones rarely found in circulating CD4^+^ T cells. J Virol. (2006) 80:6441–57. 10.1128/JVI.00591-06 16775332 PMC1488985

[28] Tobin NH Learn GH Holte SE Wang Y Melvin AJ McKernan JL. Evidence that low-level viremias during effective highly active antiretroviral therapy result from two processes: expression of archival virus and replication of virus. J Virol. (2005) 79:9625–34. 10.1128/JVI.79.15.9625-9634.2005 16014925 PMC1181593

[29] Schroder AR Shinn P Chen H Berry C Ecker JR Bushman F. HIV-1 integration in the human genome favors active genes and local hotspots. Cell. (2002) 110:521–9. 10.1016/S0092-8674(02)00864-4 12202041

[30] Symons J Cameron PU Lewin SR. HIV integration sites and implications for maintenance of the reservoir. Curr Opin HIV AIDS. (2018) 13:152–9. 10.1097/COH.0000000000000438 29206656 PMC5808998

[31] Wang Z Gurule EE Brennan TP Gerold JM Kwon KJ Hosmane NN. Expanded cellular clones carrying replication-competent HIV-1 persist, wax, and wane. Proc Natl Acad Sci USA. (2018) 115:E2575–84. 10.1073/pnas.1720665115 29483265 PMC5856552

[32] Cohn LB Silva IT Oliveira TY Rosales RA Parrish EH Learn GH. HIV-1 integration landscape during latent and active infection. Cell. (2015) 160:420–32. 10.1016/j.cell.2015.01.020 25635456 PMC4371550

[33] Maldarelli F Wu X Su L Simonetti FR Shao W Hill S. HIV latency. Specific HIV integration sites are linked to clonal expansion and persistence of infected cells. Science. (2014) 345:179–83. 10.1126/science.1254194 24968937 PMC4262401

[34] Simonetti FR Sobolewski MD Fyne E Shao W Spindler J Hattori J. Clonally expanded CD4^+^ T cells can produce infectious HIV-1 *in vivo*. Proc Natl Acad Sci USA. (2016) 113:1883–8. 10.1073/pnas.1522675113 26858442 PMC4763755

[35] Wagner TA McLaughlin S Garg K Cheung CY Larsen BB Styrchak S. HIV latency. Proliferation of cells with HIV integrated into cancer genes contributes to persistent infection. Science. (2014) 345:570–3. 10.1126/science.1256304 25011556 PMC4230336

[36] Hosmane NN Kwon KJ Bruner KM Capoferri AA Beg S Rosenbloom DI. Proliferation of latently infected CD4(+) T cells carrying replication-competent HIV-1: potential role in latent reservoir dynamics. J Exp Med. (2017) 214:959–72. 10.1084/jem.20170193 28341641 PMC5379987

[37] Bui JK Halvas EK Fyne E Sobolewski MD Koontz D Shao W. *Ex vivo* activation of CD4^+^ T-cells from donors on suppressive ART can lead to sustained production of infectious HIV-1 from a subset of infected cells. PLoS Pathog. (2017) 13:e1006230. 10.1371/journal.ppat.1006230 28225830 PMC5338860

[38] Lorenzi JC Cohen YZ Cohn LB Kreider EF Barton JP Learn GH. Paired quantitative and qualitative assessment of the replication-competent HIV-1 reservoir and comparison with integrated proviral DNA. Proc Natl Acad Sci USA. (2016) 113:E7908–16. 10.1073/pnas.1617789113 27872306 PMC5150408

[39] Ebina H Misawa N Kanemura Y Koyanagi Y. Harnessing the CRISPR/Cas9 system to disrupt latent HIV-1 provirus. Sci Rep. (2013) 3:2510. 10.1038/srep02510 23974631 PMC3752613

[40] Dash PK Kaminski R Bella R Su H Mathews S Ahooyi TM. Sequential LASER ART and CRISPR treatments eliminate HIV-1 in a subset of infected humanized mice. Nat Commun. (2019) 10:2753. 10.1038/s41467-019-10366-y 31266936 PMC6606613

[41] Deng H Liu R Ellmeier W Choe S Unutmaz D Burkhart M. Identification of a major co-receptor for primary isolates of HIV-1. Nature. (1996) 381:661–6. 10.1038/381661a0 8649511

[42] Cohen J. Likely HIV cofactor found. Science. (1996) 272:809–10. 10.1126/science.272.5263.809 8629006

[43] Biti R Ffrench R Young J Bennetts B Stewart G Liang T. HIV-1 infection in an individual homozygous for the CCR5 deletion allele. Nat Med. (1997) 3:252–3. 10.1038/nm0397-252 9055842

[44] Samson M Libert F Doranz BJ Rucker J Liesnard C Farber CM. Resistance to HIV-1 infection in caucasian individuals bearing mutant alleles of the CCR-5 chemokine receptor gene. Nature. (1996) 382:722–5. 10.1038/382722a0 8751444

[45] Scarlatti G Tresoldi E Bjorndal A Fredriksson R Colognesi C Deng HK. *In vivo* evolution of HIV-1 co-receptor usage and sensitivity to chemokine-mediated suppression. Nat Med. (1997) 3:1259–65. 10.1038/nm1197-1259 9359702

[46] Hutter G Nowak D Mossner M Ganepola S Mussig A Allers K. Long-term control of HIV by CCR5 Delta32/Delta32 stem-cell transplantation. N Engl J Med. (2009) 360:692–8. 10.1056/NEJMoa0802905 19213682

[47] Allers K Hutter G Hofmann J Loddenkemper C Rieger K Thiel E. Evidence for the cure of HIV infection by CCR5Δ32/Δ32 stem cell transplantation. Blood. (2011) 117:2791–9. 10.1182/blood-2010-09-309591 21148083

[48] Tebas P Stein D Tang WW Frank I Wang SQ Lee G. Gene editing of CCR5 in autologous CD4 T cells of persons infected with HIV. N Engl J Med. (2014) 370:901–10. 10.1056/NEJMoa1300662 24597865 PMC4084652

[49] Xu L Wang J Liu Y Xie L Su B Mou D. CRISPR-Edited stem cells in a patient with HIV and acute lymphocytic leukemia. N Engl J Med. (2019) 381:1240–7. 10.1056/NEJMoa1817426 31509667

[50] Wei X Nielsen R. CCR5-32 is deleterious in the homozygous state in humans. Nat Med. (2019) 25:909–10. 10.1038/s41591-019-0459-6 31160814 PMC6613792

[51] Mitchell RS Beitzel BF Schroder AR Shinn P Chen H Berry CC. Retroviral DNA integration: ASLV, HIV, and MLV show distinct target site preferences. PLoS Biol. (2004) 2:E234. 10.1371/journal.pbio.0020234 15314653 PMC509299

[52] Vranckx LS Demeulemeester J Saleh S Boll A Vansant G Schrijvers R. LEDGIN-mediated inhibition of integrase-LEDGF/p75 interaction reduces reactivation of residual latent HIV. EBioMedicine. (2016) 8:248–64. 10.1016/j.ebiom.2016.04.039 27428435 PMC4919729

[53] Mousseau G Clementz MA Bakeman WN Nagarsheth N Cameron M Shi J. An analog of the natural steroidal alkaloid cortistatin a potently suppresses tat-dependent HIV transcription. Cell Host Microbe. (2012) 12:97–108. 10.1016/j.chom.2012.05.016 22817991 PMC3403716

[54] Mousseau G Kessing CF Fromentin R Trautmann L Chomont N Valente ST. The tat Inhibitor didehydro-cortistatin a prevents HIV-1 reactivation from latency. MBio. (2015) 6:e00465. 10.1128/mBio.00465-15 26152583 PMC4495168

[55] Chun TW Engel D Mizell SB Hallahan CW Fischette M Park S. Effect of interleukin-2 on the pool of latently infected, resting CD4^+^ T cells in HIV-1-infected patients receiving highly active anti-retroviral therapy. Nat Med. (1999) 5:651–5. 10.1038/9498 10371503

[56] Prins JM Jurriaans S van Praag RM Blaak H van RR Schellekens PT. Immuno-activation with anti-CD3 and recombinant human IL-2 in HIV-1-infected patients on potent antiretroviral therapy. AIDS. (1999) 13:2405–10. 10.1097/00002030-199912030-00012 10597782

[57] Shan L Deng K Shroff NS Durand CM Rabi SA Yang HC. Stimulation of HIV-1-specific cytolytic T lymphocytes facilitates elimination of latent viral reservoir after virus reactivation. Immunity. (2012) 36:491–501. 10.1016/j.immuni.2012.01.014 22406268 PMC3501645

[58] Spina CA Anderson J Archin NM Bosque A Chan J Famiglietti M. An in-depth comparison of latent HIV-1 reactivation in multiple cell model systems and resting CD4^+^ T cells from aviremic patients. PLoS Pathog. (2013) 9:e1003834. 10.1371/journal.ppat.1003834 24385908 PMC3873446

[59] Archin NM Espeseth A Parker D Cheema M Hazuda D Margolis DM. Expression of latent HIV induced by the potent HDAC inhibitor suberoylanilide hydroxamic acid. AIDS Res Hum Retroviruses. (2009) 25:207–12. 10.1089/aid.2008.0191 19239360 PMC2853863

[60] Bouchat S Gatot JS Kabeya K Cardona C Colin L Herbein G. Histone methyltransferase inhibitors induce HIV-1 recovery in resting CD4(+) T cells from HIV-1-infected HAART-treated patients. AIDS. (2012) 26:1473–82. 10.1097/QAD.0b013e32835535f5 22555163

[61] Imai K Togami H Okamoto T. Involvement of histone H3 lysine 9 (H3K9) methyltransferase G9a in the maintenance of HIV-1 latency and its reactivation by BIX01294. J Biol Chem. (2010) 285:16538–45. 10.1074/jbc.M110.103531 20335163 PMC2878073

[62] Del Prete GQ Oswald K Lara A Shoemaker R Smedley J Macallister R. Elevated plasma viral loads in romidepsin-treated simian immunodeficiency virus-infected rhesus macaques on suppressive combination antiretroviral therapy. Antimicrob Agents Chemother. (2015) 60:1560–72. 10.1128/AAC.02625-15 26711758 PMC4776002

[63] Lucera MB Tilton CA Mao H Dobrowolski C Tabler CO Haqqani AA. The histone deacetylase inhibitor vorinostat (SAHA) increases the susceptibility of uninfected CD4^+^ T cells to HIV by increasing the kinetics and efficiency of postentry viral events. J Virol. (2014) 88:10803–12. 10.1128/JVI.00320-14 25008921 PMC4178860

[64] Shirakawa K Chavez L Hakre S Calvanese V Verdin E. Reactivation of latent HIV by histone deacetylase inhibitors. Trends Microbiol. (2013) 21:277–85. 10.1016/j.tim.2013.02.005 23517573 PMC3685471

[65] Wei DG Chiang V Fyne E Balakrishnan M Barnes T Graupe M. Histone deacetylase inhibitor romidepsin induces HIV expression in CD4 T cells from patients on suppressive antiretroviral therapy at concentrations achieved by clinical dosing. PLoS Pathog. (2014) 10:e1004071. 10.1371/journal.ppat.1004071 24722454 PMC3983056

[66] Demoustier A Gubler B Lambotte O de Goer MG Wallon C Goujard C. In patients on prolonged HAART, a significant pool of HIV infected CD4 T cells are HIV-specific. AIDS. (2002) 16:1749–54. 10.1097/00002030-200209060-00006 12218385

[67] Douek DC Betts MR Brenchley JM Hill BJ Ambrozak DR Ngai KL. A novel approach to the analysis of specificity, clonality, and frequency of HIV-specific T cell responses reveals a potential mechanism for control of viral escape. J Immunol. (2002) 168:3099–104. 10.4049/jimmunol.168.6.3099 11884484

[68] Pankrac J Klein K Mann JFS. Eradication of HIV-1 latent reservoirs through therapeutic vaccination. AIDS Res Ther. (2017) 14:45. 10.1186/s12981-017-0177-4 28893280 PMC5594457

[69] Tsai A Irrinki A Kaur J Cihlar T Kukolj G Sloan DD. Toll-like receptor 7 agonist GS-9620 induces HIV expression and HIV-specific immunity in cells from HIV-infected individuals on suppressive antiretroviral therapy. J Virol. (2017) 91:e02166–16. 10.1128/JVI.02166-16 28179531 PMC5375698

[70] Bullen CK Laird GM Durand CM Siliciano JD Siliciano RF. New *ex vivo* approaches distinguish effective and ineffective single agents for reversing HIV-1 latency *in vivo*. Nat Med. (2014) 20:425–9. 10.1038/nm.3489 24658076 PMC3981911

[71] Sogaard OS Graversen ME Leth S Olesen R Brinkmann CR Nissen SK. The depsipeptide romidepsin reverses HIV-1 latency *in vivo*. PLoS Pathog. (2015) 11:e1005142. 10.1371/journal.ppat.1005142 26379282 PMC4575032

[72] Narasimhan S Coumou J Schuijt TJ Boder E Hovius JW Fikrig E. A tick gut protein with fibronectin III domains aids *Borrelia burgdorferi* congregation to the gut during transmission. PLoS Pathog. (2014) 10:e1004278. 10.1371/journal.ppat.1004278 25102051 PMC4125277

[73] Waibel M Christiansen AJ Hibbs ML Shortt J Jones SA Simpson I. Manipulation of B-cell responses with histone deacetylase inhibitors. Nat Commun. (2015) 6:6838. 10.1038/ncomms7838 25913720

[74] Barouch DH Deeks SG. Immunologic strategies for HIV-1 remission and eradication. Science. (2014) 345:169–74. 10.1126/science.1255512 25013067 PMC4096716

[75] Gross G Waks T Eshhar Z. Expression of immunoglobulin-T-cell receptor chimeric molecules as functional receptors with antibody-type specificity. Proc Natl Acad Sci USA. (1989) 86:10024–8. 10.1073/pnas.86.24.10024 2513569 PMC298636

[76] Ramos CA Dotti G. Chimeric antigen receptor (CAR)-engineered lymphocytes for cancer therapy. Expert Opin Biol Ther. (2011) 11:855–73. 10.1517/14712598.2011.573476 21463133 PMC3107373

[77] Sadelain M Brentjens R Riviere I. The basic principles of chimeric antigen receptor design. Cancer Discov. (2013) 3:388–98. 10.1158/2159-8290.CD-12-0548 23550147 PMC3667586

[78] Zhang C Liu J Zhong JF Zhang X. Engineering CAR-T cells. Biomark Res. (2017) 5:22. 10.1186/s40364-017-0102-y 28652918 PMC5482931

[79] Finney HM Lawson AD Bebbington CR Weir AN. Chimeric receptors providing both primary and costimulatory signaling in T cells from a single gene product. J Immunol. (1998) 161:2791–7. 9743337

[80] Krause A Guo HF Latouche JB Tan C Cheung NK Sadelain M. Antigen-dependent CD28 signaling selectively enhances survival and proliferation in genetically modified activated human primary T lymphocytes. J Exp Med. (1998) 188:619–26. 10.1084/jem.188.4.619 9705944 PMC2213361

[81] Milone MC Fish JD Carpenito C Carroll RG Binder GK Teachey D. Chimeric receptors containing CD137 signal transduction domains mediate enhanced survival of T cells and increased antileukemic efficacy *in vivo*. Mol Ther. (2009) 17:1453–64. 10.1038/mt.2009.83 19384291 PMC2805264

[82] Tammana S Huang X Wong M Milone MC Ma L Levine BL. 4-1BB and CD28 signaling plays a synergistic role in redirecting umbilical cord blood T cells against B-cell malignancies. Hum Gene Ther. (2010) 21:75–86. 10.1089/hum.2009.122 19719389 PMC2861957

[83] Chmielewski M Abken H. TRUCKs: the fourth generation of CARs. Expert Opin Biol Ther. (2015) 15:1145–54. 10.1517/14712598.2015.1046430 25985798

[84] Porter DL Levine BL Kalos M Bagg A June CH. Chimeric antigen receptor-modified T cells in chronic lymphoid leukemia. N Engl J Med. (2011) 365:725–33. 10.1056/NEJMoa1103849 21830940 PMC3387277

[85] Maude SL Frey N Shaw PA Aplenc R Barrett DM Bunin NJ. Chimeric antigen receptor T cells for sustained remissions in leukemia. N Engl J Med. (2014) 371:1507–17. 10.1056/NEJMoa1407222 25317870 PMC4267531

[86] Schuster SJ Svoboda J Chong EA Nasta SD Mato AR Anak O. Chimeric antigen receptor T cells in refractory B-Cell lymphomas. N Engl J Med. (2017) 377:2545–54. 10.1056/NEJMoa1708566 29226764 PMC5788566

[87] Newick K O'Brien S Moon E Albelda SM. CAR T cell therapy for solid tumors. Annu Rev Med. (2017) 68:139–52. 10.1146/annurev-med-062315-120245 27860544

[88] Kalos M Levine BL Porter DL Katz S Grupp SA Bagg A. T cells with chimeric antigen receptors have potent antitumor effects and can establish memory in patients with advanced leukemia. Sci Transl Med. (2011) 3:95ra73. 10.1126/scitranslmed.3002842 21832238 PMC3393096

[89] Schmitz JE Kuroda MJ Santra S Sasseville VG Simon MA Lifton MA. Control of viremia in simian immunodeficiency virus infection by CD8^+^ lymphocytes. Science. (1999) 283:857–60. 10.1126/science.283.5403.857 9933172

[90] Okulicz JF Lambotte O. Epidemiology and clinical characteristics of elite controllers. Curr Opin HIV AIDS. (2011) 6:163–8. 10.1097/COH.0b013e328344f35e 21502920

[91] Loffredo JT Sidney J Bean AT Beal DR Bardet W Wahl A. Two MHC class I molecules associated with elite control of immunodeficiency virus replication, Mamu-B^*^08 and HLA-B^*^2705, bind peptides with sequence similarity. J Immunol. (2009) 182:7763–75. 10.4049/jimmunol.0900111 19494300 PMC2701622

[92] Saez-Cirion A Lacabaratz C Lambotte O Versmisse P Urrutia A Boufassa F. HIV controllers exhibit potent CD8 T cell capacity to suppress HIV infection *ex vivo* and peculiar cytotoxic T lymphocyte activation phenotype. Proc Natl Acad Sci USA. (2007) 104:6776–81. 10.1073/pnas.0611244104 17428922 PMC1851664

[93] Borrow P Lewicki H Wei X Horwitz MS Peffer N Meyers H. Antiviral pressure exerted by HIV-1-specific cytotoxic T lymphocytes (CTLs) during primary infection demonstrated by rapid selection of CTL escape virus. Nat Med. (1997) 3:205–11. 10.1038/nm0297-205 9018240

[94] Koup RA Safrit JT Cao Y Andrews CA McLeod G Borkowsky W. Temporal association of cellular immune responses with the initial control of viremia in primary human immunodeficiency virus type 1 syndrome. J Virol. (1994) 68:4650–5. 10.1128/JVI.68.7.4650-4655.1994 8207839 PMC236393

[95] Deng K Pertea M Rongvaux A Wang L Durand CM Ghiaur G. Broad CTL response is required to clear latent HIV-1 due to dominance of escape mutations. Nature. (2015) 517:381–5. 10.1038/nature14053 25561180 PMC4406054

[96] Mothe B Llano A Ibarrondo J Zamarreno J Schiaulini M Miranda C. CTL responses of high functional avidity and broad variant cross-reactivity are associated with HIV control. PLoS ONE. (2012) 7:e29717. 10.1371/journal.pone.0029717 22238642 PMC3251596

[97] Deeks SG Wagner B Anton PA Mitsuyasu RT Scadden DT Huang C. A phase II randomized study of HIV-specific T-cell gene therapy in subjects with undetectable plasma viremia on combination antiretroviral therapy. Mol Ther. (2002) 5:788–97. 10.1006/mthe.2002.0611 12027564

[98] Ali A Kitchen SG Chen ISY Ng HL Zack JA Yang OO. HIV-1-specific chimeric antigen receptors based on broadly neutralizing antibodies. J Virol. (2016) 90:6999–7006. 10.1128/JVI.00805-16 27226366 PMC4944295

[99] Hale M Mesojednik T Romano Ibarra GS Sahni J Bernard A Sommer K. Engineering HIV-resistant, anti-HIV chimeric antigen receptor T cells. Mol Ther. (2017) 25:570–9. 10.1016/j.ymthe.2016.12.023 28143740 PMC5363191

[100] Yang OO Tran AC Kalams SA Johnson RP Roberts MR Walker BD. Lysis of HIV-1-infected cells and inhibition of viral replication by universal receptor T cells. Proc Natl Acad Sci USA. (1997) 94:11478–83. 10.1073/pnas.94.21.11478 9326635 PMC23511

[101] Kwong PD Mascola JR Nabel GJ. Broadly neutralizing antibodies and the search for an HIV-1 vaccine: the end of the beginning. Nat Rev Immunol. (2013) 13:693–701. 10.1038/nri3516 23969737

[102] Collins KL Chen BK Kalams SA Walker BD Baltimore D. HIV-1 Nef protein protects infected primary cells against killing by cytotoxic T lymphocytes. Nature. (1998) 391:397–401. 10.1038/34929 9450757

[103] Schwartz O Marechal V Le GS Lemonnier F Heard JM. Endocytosis of major histocompatibility complex class I molecules is induced by the HIV-1 Nef protein. Nat Med. (1996) 2:338–42. 10.1038/nm0396-338 8612235

[104] Lipowska-Bhalla G Gilham DE Hawkins RE Rothwell DG. Targeted immunotherapy of cancer with CAR T cells: achievements and challenges. Cancer Immunol Immunother. (2012) 61:953–62. 10.1007/s00262-012-1254-0 22527245 PMC11028843

[105] Scholler J Brady TL Binder-Scholl G Hwang WT Plesa G Hege KM. Decade-long safety and function of retroviral-modified chimeric antigen receptor T cells. Sci Transl Med. (2012) 4:132ra53. 10.1126/scitranslmed.3003761 22553251 PMC4368443

[106] Grupp SA Kalos M Barrett D Aplenc R Porter DL Rheingold SR. Chimeric antigen receptor-modified T cells for acute lymphoid leukemia. N Engl J Med. (2013) 368:1509–18. 10.1056/NEJMoa1215134 23527958 PMC4058440

[107] Thompson KA Cherry CL Bell JE McLean CA. Brain cell reservoirs of latent virus in presymptomatic HIV-infected individuals. Am J Pathol. (2011) 179:1623–9. 10.1016/j.ajpath.2011.06.039 21871429 PMC3181362

[108] Anthony-Gonda K Bardhi A Ray A Flerin N Li M Chen W. Multispecific anti-HIV duoCAR-T cells display broad *in vitro* antiviral activity and potent *in vivo* elimination of HIV-infected cells in a humanized mouse model. Sci Transl Med. (2019) 11:eaav5685. 10.1126/scitranslmed.aav5685 31391322 PMC7136029

[109] Mitsuyasu RT Anton PA Deeks SG Scadden DT Connick E Downs MT. Prolonged survival and tissue trafficking following adoptive transfer of CD4zeta gene-modified autologous CD4^+^ and CD8^+^ T cells in human immunodeficiency virus-infected subjects. Blood. (2000) 96:785–93. 10.1182/blood.V96.3.785 10910888

[110] Brodie SJ Lewinsohn DA Patterson BK Jiyamapa D Krieger J Corey L. *In vivo* migration and function of transferred HIV-1-specific cytotoxic T cells. Nat Med. (1999) 5:34–41. 10.1038/4716 9883837

[111] Riddell SR Elliott M Lewinsohn DA Gilbert MJ Wilson L Manley SA. T-cell mediated rejection of gene-modified HIV-specific cytotoxic T lymphocytes in HIV-infected patients. Nat Med. (1996) 2:216–23. 10.1038/nm0296-216 8574968

[112] Tan R Xu X Ogg GS Hansasuta P Dong T Rostron T. Rapid death of adoptively transferred T cells in acquired immunodeficiency syndrome. Blood. (1999) 93:1506–10. 10.1182/blood.V93.5.1506 10029578

[113] Jena B Dotti G Cooper LJ. Redirecting T-cell specificity by introducing a tumor-specific chimeric antigen receptor. Blood. (2010) 116:1035–44. 10.1182/blood-2010-01-043737 20439624 PMC2938125

[114] Sadelain M Brentjens R Riviere I. The promise and potential pitfalls of chimeric antigen receptors. Curr Opin Immunol. (2009) 21:215–23. 10.1016/j.coi.2009.02.009 19327974 PMC5548385

[115] Sockolosky JT Trotta E Parisi G Picton L Su LL Le AC. Selective targeting of engineered T cells using orthogonal IL-2 cytokine-receptor complexes. Science. (2018) 359:1037–42. 10.1126/science.aar3246 29496879 PMC5947856

[116] Eyquem J Mansilla-Soto J Giavridis T van der Stegen SJ Hamieh M Cunanan KM. Targeting a CAR to the TRAC locus with CRISPR/Cas9 enhances tumour rejection. Nature. (2017) 543:113–7. 10.1038/nature21405 28225754 PMC5558614

[117] Fraietta JA Nobles CL Sammons MA Lundh S Carty SA Reich TJ. Disruption of TET2 promotes the therapeutic efficacy of CD19-targeted T cells. Nature. (2018) 558:307–12. 10.1038/s41586-018-0178-z 29849141 PMC6320248

[118] Bitton N Verrier F Debre P Gorochov G. Characterization of T cell-expressed chimeric receptors with antibody-type specificity for the CD4 binding site of HIV-1 gp120. Eur J Immunol. (1998) 28:4177–87. 10.1002/(SICI)1521-4141(199812)28:12<4177::AID-IMMU4177>3.0.CO;2-J 9862354

[119] Brenchley JM Schacker TW Ruff LE Price DA Taylor JH Beilman GJ. CD4^+^ T cell depletion during all stages of HIV disease occurs predominantly in the gastrointestinal tract. J Exp Med. (2004) 200:749–59. 10.1084/jem.20040874 15365096 PMC2211962

[120] Kamata M Kim PY Ng HL Ringpis GE Kranz E Chan J. Ectopic expression of anti-HIV-1 shRNAs protects CD8(+) T cells modified with CD4zeta CAR from HIV-1 infection and alleviates impairment of cell proliferation. Biochem Biophys Res Commun. (2015) 463:216–21. 10.1016/j.bbrc.2015.05.026 25998390 PMC4686265

[121] Ghanem MH Bolivar-Wagers S Dey B Hajduczki A Vargas-Inchaustegui DA Danielson DT. Bispecific chimeric antigen receptors targeting the CD4 binding site and high-mannose glycans of gp120 optimized for anti-human immunodeficiency virus potency and breadth with minimal immunogenicity. Cytotherapy. (2018) 20:407–19. 10.1016/j.jcyt.2017.11.001 29306566

[122] Romeo C Seed B. Cellular immunity to HIV activated by CD4 fused to T cell or Fc receptor polypeptides. Cell. (1991) 64:1037–46. 10.1016/0092-8674(91)90327-U 1900456

[123] Turtle CJ Hanafi LA Berger C Gooley TA Cherian S Hudecek M. CD19 CAR-T cells of defined CD4(+): CD8(+) composition in adult B cell ALL patients. J Clin Invest. (2016) 126:2123–38. 10.1172/JCI85309 27111235 PMC4887159

[124] Gust J Hay KA Hanafi LA Li D Myerson D Gonzalez-Cuyar LF. Endothelial activation and blood-brain barrier disruption in neurotoxicity after adoptive immunotherapy with CD19 CAR-T Cells. Cancer Discov. (2017) 7:1404–19. 10.1158/2159-8290.CD-17-0698 29025771 PMC5718945

[125] Neelapu S Locke F Bartlett N Lekakis L Miklos D Jacobson C. Axicabtagene ciloleucel CAR T-cell therapy in refractory large B-cell lymphoma. N Engl J Med. (2017) 377:2531–44. 10.1056/NEJMoa1707447 29226797 PMC5882485

[126] Lee D Gardner R Porter D. Current concepts in the diagnosis and management of cytokine release syndrome. Blood. (2016) 128:1533. 10.1182/blood-2016-07-730689 24876563 PMC4093680

[127] Neelapu SS Tummala S Kebriaei P Wierda W Gutierrez C Locke FL. Chimeric antigen receptor T-cell therapy - assessment and management of toxicities. Nat Rev Clin Oncol. (2018) 15:47–62. 10.1038/nrclinonc.2017.148 28925994 PMC6733403

[128] Leibman RS Richardson MW Ellebrecht CT Maldini CR Glover JA Secreto AJ. Supraphysiologic control over HIV-1 replication mediated by CD8 T cells expressing a re-engineered CD4-based chimeric antigen receptor. Plos Pathog. (2017) 13:e1006613. 10.1371/journal.ppat.1006613 29023549 PMC5638568

[129] Haran KP Hajduczki A Pampusch MS Mwakalundwa G Vargas-Inchaustegui DA Rakasz EG. Simian immunodeficiency virus (SIV)-specific chimeric antigen receptor-T cell engineered to target B cell follicles and suppress SIV replication. Front Immunol. (2018) 9:492. 10.3389/fimmu.2018.00492 29616024 PMC5869724

[130] Wu CY Roybal KT Puchner EM Onuffer J Lim WA. Remote control of therapeutic T cells through a small molecule-gated chimeric receptor. Science. (2015) 350:aab4077. 10.1126/science.aab4077 26405231 PMC4721629

[131] Torikai H Reik A Liu PQ Zhou Y Zhang L Maiti S. A foundation for universal T-cell based immunotherapy: T cells engineered to express a CD19-specific chimeric-antigen-receptor and eliminate expression of endogenous TCR. Blood. (2012) 119:5697–705. 10.1182/blood-2012-01-405365 22535661 PMC3382929

[132] Hale M Lee B Honaker Y Leung WH Grier AE Jacobs HM. Homology-directed recombination for enhanced engineering of chimeric antigen receptor T cells. Mol Ther Methods Clin Dev. (2017) 4:192–203. 10.1016/j.omtm.2016.12.008 28345004 PMC5363294

[133] Liu D Tian S Zhang K Xiong W Lubaki NM Chen Z. Chimeric antigen receptor (CAR)-modified natural killer cell-based immunotherapy and immunological synapse formation in cancer and HIV. Protein Cell. (2017) 8:861–77. 10.1007/s13238-017-0415-5 28488245 PMC5712291

[134] Zhen A Kamata M Rezek V Rick J Levin B Kasparian S. HIV-specific immunity derived from chimeric antigen receptor-engineered stem cells. Mol Ther. (2015) 23:1358–67. 10.1038/mt.2015.102 26050990 PMC4817874

